# Is the staple diet eaten in Medawachchiya, Sri Lanka, a predisposing factor in the development of chronic kidney disease of unknown etiology? - A comparison based on urinary β_2_-microglobulin measurements

**DOI:** 10.1186/1471-2369-15-103

**Published:** 2014-07-02

**Authors:** EA Ranga IE Siriwardhana, Ponnumperuma AJ Perera, Ramiah Sivakanesan, Thilak Abeysekara, Danaseela B Nugegoda, Kosala GAD Weerakoon

**Affiliations:** 1 Department of Biochemistry, Faculty of Medicine and Allied Sciences, Rajarata University of Sri Lanka, Saliyapura, Anuradhapura, Sri Lanka; 2 Department of Biochemistry, Faculty of Medicine, University of Peradeniya, Peradeniya, Sri Lanka; 3 Department of Pharmacology, Faculty of Medicine, University of Peradeniya, Peradeniya, Sri Lanka; 4 Department of Community Medicine, SAITM Faculty of Medicine, Malabe, Sri Lanka; 5 Department of Parasitology, Faculty of Medicine and Allied Sciences, Rajarata University of Sri Lanka, Saliyapura, Anuradhapura, Sri Lanka; 6 Postgraduate Institute of Science, University of Peradeniya, Peradeniya, Sri Lanka

**Keywords:** Chronic kidney disease, Heavy metals, Dietary patterns, Urinary β_2_ microglobulin

## Abstract

**Background:**

Exact mechanism of causation of chronic kidney disease of unknown etiology (CKDu) in Sri Lanka is not described to date, despite the identification of possible multiple risk factors. Questions have been raised as to why only some are affected while others remain intact, though they are inhabitants of the same locality.

**Methods:**

Comparative studies were carried out, assessing urinary β_2_ microglobulin (β_2_m) and the dietary patterns of CKDu patients and age sex matched non-CKDu subjects. Urinary β2m levels of spot urine samples were analyzed using the Enzyme-linked Immunosorbent assay (ELISA) and dietary patterns were studied using twenty four hour dietary recalls and frequency consumption of foods of animal origin performed on three occasions at six months intervals within a period of one and half years.

**Results:**

The mean urinary β_2_m level of CKDu patients from Medawachchiya was significantly (p < 0.05) higher when compared with that of the non-CKDu subjects. The mean urinary β_2_m level of the non-CKDu subjects was within the reference limits for spot urine samples (0 – 0.3 μg/mL). White raw rice was the staple diet of both CKDu patients and non-CKDu subjects and the level of consumption was almost the same. The consumption of fresh water fish products of CKDu patients under high (14, 14%), moderate (36, 36%), low (26, 26%) and less (20, 20%) categories did not show significant variations (p > 0.05) compared to non-CKDu subjects.

**Conclusions:**

Staple food in diet and the consumption pattern of CKDu patients from Medawachchiya were similar to that of non-CKDu subjects from the same area despite their urinary β_2_m concentration being significantly higher.

## Background

Chronic kidney disease of unknown aetiology (CKDu) has been reported in developing countries including Nicaragua, countries of Balkan region, Tunisia and Sri Lanka [1-3]. CKDu has been reported from certain parts of Sri Lanka, including the North Central, North Western and Uva provinces and the prevalence is highest in the North Central Province. The affected areas belong to the dry and intermediate climatic zones of Sri Lanka. The majority of the CKDu affected inhabitants in these areas, belong to the low –socioeconomic farming community. The prior studies indicated the affected populations to predominantly be young males, whilst the more recent studies have shown high prevalence among females and more severe stages of CKDu among males [4,5].

Numerous studies of diverse disciplines have been carried out to reveal the credible contributory factors for the disease. The prevalence of CKDu, epidemiological studies and environmental risk factors were meticulously scrutinized in these studies. The scientific correlation between the incidence of CKDu and the suggested risk factors still remain detached.

Urinary β_2_ microglobulin (β_2_m) has been used as a marker of heavy metal poisoning that includes cadmium, arsenic and lead [6]. Current research conducted on CKDu in Sri Lanka suggests heavy metals as the major aetiological factor and food chains as the possible route of heavy metal entry into the bodies of those afflicted by the disease [5,7]. When the endemic areas are concerned, non-affected individuals reside in the same vicinity and household as affected for long periods of time without being affected by CKDu and studying different aspects of lifestyle, occupations, dietary patterns etc. of these two groups would be much intriguing. Main objectives of the current study were to compare the variation in urinary β_2_m excretion of CKDu patients and non-CKDu subjects, inhabiting in a CKDu endemic area, and to compare the dietary patterns of CKDu patients and non-CKDu subjects from the same area.

## Methods

### Analysis of urinary β_2_m levels of CKDu patients and non-CKDu subjects

Thirty CKDu patients, who had been diagnosed as having CKDu subsequent to medical and biochemical examinations by a consultant nephrologist, and residing in Medawachchiya divisional secretariat were randomly selected. Presence of proteinuria, elevated levels of serum creatinine (>1.2 mg/dL) and confirmed abdominal ultrasound scan /renal biopsy reports were used as the inclusion criteria for selecting CKDu patients. All known causes of CKD including diabetes mellitus, long standing hypertension, glomerular nephritis, urolithaisis, and congenital kidney diseases and having a past history of snake bite and leptospirosis were excluded from the study. Age sex matched non-CKDu subjects (n = 30) residing in the same areas were recruited subsequent to clinical and biochemical examinations by a consultant nephrologist. The subjects having normal levels for the tested parameters and comply with all the exclusion criteria of the CKDu patients were selected as non-CKDu subjects. All the non-CKDu subjects had serum creatinine and random blood glucose levels below 1.0 mg/dL and 140 mg/dL respectively, blood pressure values were ≤ 120/80 mmHg and were negative for proteinuria.

Subjects of both groups were made aware of the research study and their written informed consent was obtained prior to their participation. Spot urine samples were obtained into sterilized polypropylene urine containers (30 mL), pH was adjusted to > 6, aliquoted and immediately transported to the laboratory in ice boxes and stored at −40˚C till the analysis is done. The samples were analyzed for urinary β_2_m in duplicate using an ELISA test kit (BIOQUANT, B-2MG BQO10T, Germany). The normality of the urinary β_2_m values obtained was confirmed with tests for normality including Anderson-Darling, Ryan-Joiner and Kolmogorov-Smirnov and analyzed using parametric and non parametric tests in Minitab version 16.0 (http://www.minitab.com/en-us/).

### Dietary study

The urinary β_2_m study was followed by a comparative dietary investigation which included CKDu patients (n = 100; 59 males and 41 females) and age sex matched non-CKDu subjects (n = 100; 59 males and 41 females). The CKDu patients were randomly selected from the pool of CKDu patients attending the renal care unit of the Medawachchiya base hospital and were inhabitants of the Medewachchiya divisional secretariat administrative unit. The non-CKDu subjects, confirmed of not having CKDu, subsequent to clinical examination and testing their blood and urine samples, were drawn randomly from the same inhabitant areas as CKDu patients. The inclusion and exclusion criteria of CKDu patients and non-CKDu subjects were comparable to that of the Urinary β_2_m study mentioned above. The CKDu patients were in stage II (n = 16), stage III (n = 58), stage IV (n = 24) and stage V (n = 2) as per National Kidney Foundation, Kidney disease outcomes quality initiative, guidelines. All subjects were informed about the aims and objectives of the study, and their written consent was obtained prior to their participation.

Twenty four hour dietary recalls and food frequency questionnaires [8,9] were used to compare the dietary patterns of the two groups. Data into 24 hour dietary recalls were collected at three occasions from each individual at 6 months intervals within a period of one and half years, with the objective of detecting possible variations in the dietary patterns, during different periods of the year. The consumption of foods of animal origin was assessed using a food frequency questionnaire, that focused on consumption of the four main types of animal foods, namely fresh fish (fresh water and sea), dry fish (fresh water, sea, small and large), egg and meat (chicken, pork, beef, mutton and other wild types). Frequency was recorded as more than five times per week, less than or equal to five times per week, once a fortnight or less than that [9]. Data collected were analyzed using Minitab version 16.0 (http://www.minitab.com/en-us/). Chi-square tests, Fisher’s exact tests and Odds ratio calculation were used for analysis. Confidence intervals (95%) were calculated for the Odds ratios.

Ethical clearance for the study was obtained from the “Research, Ethical Review and Higher Degrees Committee” of the Faculty of Medicine, University of Peradeniya, Sri Lanka. The administrative clearance for the study was obtained from the Regional Director of Health Services of Anuradhapura, and the District Medical Officer, Medawachchiya hospital.

## Results

### Urinary β_2_m

The mean urinary β_2_m level of CKDu patients (1.24 ± 0.71 μg/mL) from Medawachchiya was significantly (p < 0.05) higher than that of non-CKDu subjects (0.16 ± 0.05 μg/mL) (Table 1). The mean urinary β_2_m level of the non-CKDu subjects was within the reference limits for spot urine samples (0–0.3 μg/mL).

**Table 1:** **Variation of urinary β**_**2**_**m levels among CKDu Patients and non-CKDu subjects of the urinary β**_**2**_**m study**

**Parameter**	**CKDu patients**	**Non-CKDu subjects**	**P value**
	**(n = 30)**	**(n = 30)**	
Male : Female	3:2	3:2	-
Age(years; Median, Mean ± SD)	47.0, 46.3 ± 5.9	47.0, 46.7 ± 9.4	0.948
Male	46.3 ± 5.6	49.0 ± 10.4	0.743
Female	46.3 ± 6.6	43.2 ± 6.6	0.800
Urinary β_2_m(μg/mL, Median, Mean ± SD)	1.17, 1.24 ± 0.71	0.15, 0.16 ± 0.05	< 0.001
Male	1.22, 1.31 ± 0.76	0.14, 0.14 ± 0.04	< 0.005
Female	1.07, 1.14 ± 0.65	0.16, 0.17 ± 0.05	< 0.005

β2m = β2-microglobulin, SD = standard deviation.

The mean urinary β_2_m levels of CKDu patients gradually increased with the degree of deterioration of kidney function (Table 2).

**Table 2:** **Mean urinary β**_**2**_**m concentration of CKDu patients against the stage of CKD as per K/DOQI classification**

**Stage of CKD**	**Number of CKDu patients**	**GFR (mL/min/1.73 m**^**2**^**)**	**β**_**2**_**m (μg/mL) (Median, Mean ± SD)**
1	0	≥ 90	-
2	3	60 – 89	0.428, 0.603 ± 0.35
3	12	30 – 59	1.034, 0.950 ± 0.43
4	13	15 – 29	1.547, 1.537 ± 0.75
5	2	< 15	2.046, 2.046 ± 0.91

K/DOQI = Kidney disease outcomes quality initiative, CKD = Chronic kidney disease, GFR = Glomerular filtration rate.

### Dietary study

Pertaining to the dietary study, anthropometric measurements were taken at the commencement of the study and the mean BMI values of the non-CKDu subjects was closer to the upper limit of normal BMI levels for Asians (18.5–23.0 kg m^−2^), whilst that of CKDu patients was closer to lower limit of normal (Table 3).

**Table 3:** CKDu patients and non-CKDu subjects of the dietary study by age, sex and BMI categories

**Variable**	**CKDu patients**	**Non-CKDu subjects**	**P value**
	**(n = 100)**	**(n = 100)**	
Age (years, Mean ± SD)	47.8 ± 9.6	47.7 ± 9.2	0.922
Height (cm, Mean ± SD)	164.2 ± 7.9	159.2 ± 7.7	< 0.001
Weight (kg, Mean ± SD)	56.9 ± 7.4	60.7 ± 8.9	0.001
BMI (kg m^−2^, Mean ± SD)	21.3 ± 3.3	23.9 ± 2.9	< 0.001

For CKDu patients: n = 100; 59 males and 41 females, for non-CKDu subjects: n = 100; 59 males and 41 females.

All subjects of the dietary study, in both groups generally consumed 3 main meals per day, namely breakfast, lunch and dinner and their staple food was rice. Majority of meals reported in both groups comprised of rice and rice flour products (Table 4). Consumption of bread/wheat based products and rice flour products was low in both groups and pulses been the least consumed food variety.

**Table 4:** Staple food types reported in the dietary study among CKDu patients and non-CKDu subjects

**Cereal type**	**CKDu patients**	**Non-CKDu subjects**	**χ2**	**Odds ratio**
	**No. of meals reported %**	**No. of meals reported %**	**(p value)**	**(95% CI)**
	**(N)**	**(N)**		
Rice and rice products	91.6 (826)	96.3 (846)	20.009 (0.007)	0.518 (0.319 – 0.842)
Millet	1.9 (17)	0.2 (2)	0.026*	-
Pulses and seeds	1.0 (9)	0.6 (5)	0.006 (0.940)	0.955 (0.290 – 3.146)
Bread/wheat products**	5.5 (49)	2.9 (26)	-	-

(N) = Number of meals.

χ2 > 1: Presence of an association (when calculated considering wheat as the reference food).

p < 0.05: Association is significant.

CI: 95% confidence interval.

*Fisher’s exact test has been used.

Consumption of staple food types were recorded in 901 and 879 meals of CKDu patients (n = 100) and non-CKDu subjects (n = 100), respectively.

**Reference food.

**Figure 1:**
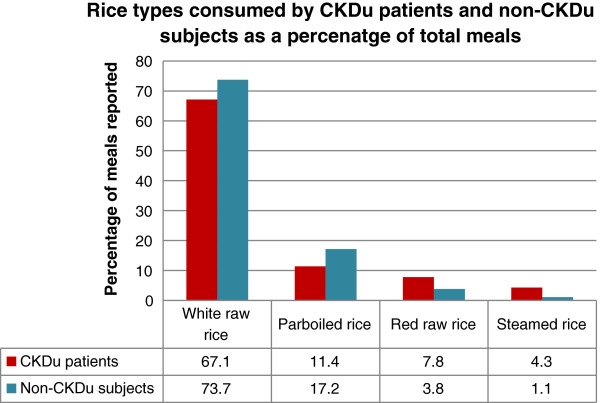
**Different rice types consumed by CKDu patients and non-CKDu subjects.** Rice types were recorded from 901 and 879 meals of CKDu patients (n = 100) and non-CKDu subjects (n = 100), respectively.

In the statistical analysis of dietary data, one food type of each section, which was not locally grown, was considered as the reference food. The selection of the reference food was based on the assumption that this particular food by means of food chain had the least probability of contributing towards the development of CKDu. The effect of other food categories on the development CKDu was compared with that of the reference food. Significantly high (p < 0.05) consumption of rice and rice products (OR: 0.5181, CI: 0.3189–0.8415) and significantly low consumption of millet products (OR: 4.5102, CI: 0.9665–21.0473) were observed when analyzed considering bread and wheat flour products as the reference food (Table 4).White raw rice was commonly consumed by both CKDu patients (67.15%, n = 605 out of 901 meals) and non-CKDu subjects (73.7%, n = 648 out of 879 meals). Parboiled rice followed by red raw rice and steamed rice were the other rice types consumed by both groups (Figure 1).

The accompaniments of the main meals were identified under 8 main categories including animal sources, fruit vegetables, pulses & products, starchy vegetables, sambol/kirihodi, other vegetable accompaniments, green leaves, and leguminous pods (Table 5). Animal sources were the most common accompaniment of the meals in both groups. The animal sources included different varieties of fish, meat and eggs. Fruit vegetables, recorded as the second common accompaniment among CKDu patients, included 19 types of vegetables: brinjal (*Solanum melongena*), snake gourd (*Trichosanthes anguina*), ridge gourd (*Luffa acutangula*), tomato (*Lycopersicum esculentum*), eggplant (*Solanum melongena*) etc. Brinjal was the type commonly consumed by both groups, 5.5% for CKDu patients and 5.6% for non-CKDu subjects. The other favorite fruit vegetables eaten among the CKDu patients were, cucumber (*Cucumis sativus*) (3.6%), snake gourd (2.7%) and drumsticks (*Moringa olifera*) (2.6%) whilst that of non-CKDu subjects were kekiri (*Cucumis melo*) (2.3%), tomato (2.3%), capsicum (2.2%) and drumsticks (2.1%). The consumption of curries of tamarind (*Tamarindus indica*) (0.7% in CKDu patients and 0.5% in non-CKDu subjects) and bilin (*Averrhoea bilimbi*) (0.3% in CKDu patients and 1.4% in non-CKDu subjects) was also recorded. Most of the vegetables included under fruit vegetables category were found to be grown locally. These types were told to be either grown naturally or grown manually in their home gardens or “chena”.

**Table 5:** Accompaniments in the meals consumed by the CKDu patients and non-CKDu subjects

**Category no.**	**Accompaniment in the main meal**	**CKDu patients**		**Non-CKDu subjects**		**χ**^**2**^**(p value)**	**Odds ratio (95% CI)**
		**No. of meals reported % (N)**	**No. of subjects % (n)**	**No. of meals reported % (N)**	**No. of subjects % (n)**		
1	Animal sources	35.7 (322)	97 (97)	42.1(370)	88 (88)	20.244 (<0.001)	0.5270.398-0.698
2	Fruit vegetables	33.7 (304)	97 (97)	28.3 (249)	98 (98)	4.151 (0.042)	0.7390.553-0.989
3	Pulses & products	27.8 (250)	97 (97)	28.9 (254)	95 (95)	11.899 (0.001)	0.5960.444-0.801
4	Starchy vegetables	21.9 (197)	88 (88)	11.3 (99)	69 (69)	1.164 (0.281)	1.2050.859-1.691
5	Sambol/kirihodi	21.4 (193)	89 (89)	14.4 (127)	80 (80)	0.249 (0.618)	0.9200.664-1.276
6	Other vegetable accompaniments*	20.0 (180)	86 (86)	12.4 (109)	78 (78)	-	-
7	Green leaves	16.3 (147)	83 (83)	15.8 (139)	81 (81)	6.944 (0.008)	0.6400.459-0.893
8	Leguminous pods	10.3 (93)	63 (63)	6.4 (56)	49 (49)	0.001 (0.978)	1.0060.669-1.512

(N) = Number of meals, (n) = Number of subjects.

χ2 > 1: Presence of an association (when calculated considering other vegetable accompaniments as the reference).

p < 0.05: Association is significant.

CI: 95% confidence interval.

Odds ratio > 1: Probability of the number of times the person is at risk in developing CKDu.

Accompaniments in meals were recorded from 901 meals among CKDu patients and 897 of meals among the non-CKDu subjects. *Reference food type.

Lentils, soya meat and green grams and chick pea were included under pulses and products and their consumption in the two groups was almost equal. Out of these, lentil was mostly consumed by CKDu patients as well as non-CKDu subjects. Lentils are not locally grown, and further soya products were not locally produced. Cowpea and green grams, were grown by local farmers in small as well as in large scale. During the current dietary study, cowpea was not reported as an accompaniment, and green grams though reported as an accompaniment, was very low (<1%) in consumption.

Starchy vegetables included yellow pumpkin (*Cucurbita maxima*), bread fruit (*Artocarpus altilis*), jack fruit (*Artocarpus heterophyllus*), potatoes (*Solanum tuberosum*) etc. Preparations of scraped coconut and coconut milked locally termed “Sambol and kirihodi” respectively, were frequently added as an accompaniment by subjects of both groups (21.4% and 14.4% of main meals of CKDu patients and non-CKDu subjects). These accompaniments were consumed with white raw rice, either alone or in combination with some other accompaniment. Roots such as carrot (*Daucus carota*), beetroot (*Beta vulgaris*), turnip (*Brassica rapa*), stalks such as leeks (*Allium ampeloprassum*), stems such as kohila (*Lasia spinosa*) were included under other vegetable accompaniments. Percentages of meals reported with starchy vegetables, coconut preparations and other vegetable accompaniments were higher among the CKDu patients than those of the non-CKDu group. Consumption of green leaves and leguminous pods were considerably low in both groups, when compared with other types of food categories. Among the types of green leaves consumed, Sesbania was the commonest variety of green leaves consumed by both groups. Mukunuwenna (*Alternanthre sessiles*), anguna (*Dregia volubilis)* and manioc (*Manihot esculenta*) were the other commonly consumed varieties of green leaves, by the two groups. Accompaniments consumed with the meals included, locally grown (in Medawachchiya divisional secretariat and areas around it) and externally grown (in other provinces and brought to this area) victuals. All except cho-cho/chouchoute *(Sechium edule)* among the fruit vegetables which were the second common accompaniment are reported to be grown locally. All green leaves except asamodagam (*Trachyspermum roxburghianum*) and all starchy vegetables except potatoes recorded in the current study are reported to be grown locally. The majority (75%) of items included under category 6 – other vegetable accompaniments are not grown locally. When each category of accompaniment was compared with category 6 (other vegetable accompaniments), significant variations (p < 0.05) were found in the consumption of foods of animal origin, fruit vegetables, pulses and products and green leafy vegetables.

Compiling the data collected into food frequency questionnaires on consumption of foods of animal origin revealed that consumption of locally available fresh water fish was higher (**>**5 times a week or ≤ 5 times a week) among subjects of both groups (76% of CKDu patients and 62% of non-CKDu subjects, (Table 6). Locally prepared fresh water dried fish was consumed by the subjects in each group (30% of CKDu patients and 25% of the non-CKDu subjects) with their meals at a same frequency as the fresh water fish. Frequent consumption (**>**5 times a week or ≤ 5 times a week) of dry fish (sea water type) was noted among 85% of CKDu patients and 80% of non-CKDu subjects. The consumption of sea fish, eggs and meat were generally low in both groups of subjects.

**Table 6:** Percentage frequency of consumption foods of animal origin among CKDu patients and non-CKDu subjects

**Foods of animal origin**	**> 5 times a week**		**≤ 5 times a week**		**Once in 2 weeks**		**Less**	
	**CKDu patients**	**Non-CKDu subjects**	**CKDu patients**	**Non-CKDu subjects**	**CKDu patients**	**Non-CKDu subjects**	**CKDu patients**	**Non-CKDu subjects**
**Fresh water**								
Fresh water fish	14	10	62	52	11	14	7	5
Fresh water dry fish	9	2	21	23	21	18	8	3
**Sea water**								
Sea fish	1	2	29	28	34	21	18	24
**Dry fish**								
Sprats	39	33	46	47	4	2	0	0
Other dry fish	29	19	33	42	4	5	0	3
Eggs	1	0	37	40	16	12	21	14
Chicken	0	0	11	20	21	20	36	24
Beef	0	1	2	0	1	2	9	4
Pork	0	1	1	2	3	3	11	9
Wild types*	0	1	1	2	3	2	16	21

*Wild types included meat of deer and wild bore; Data was obtained from food frequency questionnaire administered to CKDu patients (n = 100) and non-CKDu subjects (n = 100).

The consumption of fresh water fish products either in the form of fresh fish or dried fish when analyzed under 4 categories including high (>5/wk), moderate (3,4,5/wk), low (1,2/wk) and less (<1/wk) (Table 7), no significant variations were obtained (p > 0.05) between the high, moderate, low and less categories of the two groups (Table 7).

**Table 7:** Variation in consumption of fresh water fish products among CKDu patients and non-CKDu subjects

**Fish type**	**Consumption/week**								**χ**^**2 **^**(p value)**
	**High**		**Moderate**		**Low**		**Very low or nil < 1/wk**		
	**>5/wk**		**3 – 5/wk**		**2 – 1/wk**				
	**CKDu patients**	**Non-CKDu subjects**	**CKDu patients**	**Non-CKDu subjects**	**CKDu patients**	**Non-CKDu subjects**	**CKDu patients**	**Non-CKDu subjects**	
Fish	14	11	36	25	26	25	20	21	1.294 (0.730)
Dry fish	8	1	4	10	18	12	29	23	0.038*

*Fisher’s exact test has been used.

## Discussion

Mean urinary β2m level of the CKDu patients of Medawachchiya was approximately 7 times higher than that of the non-CKDu subjects. The CKDu patients were medically confirmed to be otherwise normal. Almost all β_2_m filtered by the glomerular capillary membrane is reabsorbed and catabolized by the cells of the proximal tubules [10]. Therefore the elevated urinary β_2_m levels are confirmative of the tubular damage reported in the biopsy reports of CKDu patients indicating proximal tubular damage. Studies conducted in the Balkan region, where a similar clinical scenario to that of CKDu exists, have suggested urinary β_2_m as an early maker to differentiate Balkan endemic nephropathy from other forms of nephropathies, as healthy individuals from the same situ have demonstrated elevated urinary β_2_m levels [11,12]. In the current study, the urinary mean β_2_m levels of the non-CKDu subjects was within the normal reference limits (0–0.3 μg/mL) for spot urine samples (Table 1), indicating normal tubular functions despite the normal subjects living under the equivalent environmental conditions as CKDu patients. The mean urinary β_2_m levels of CKDu patients when compared against the stage of their kidney disease gradually increased with the severity of kidney damage (Table 2), probably owing to amplified tubular damage.

Chronic exposure to heavy metals is indicated as the possible cause of CKDu in Sri Lanka by most recent studies [5,7]. Urinary level of β_2_m is increased in heavy metal toxicities causing tubular damages [13]. The elevated urinary β_2_m values obtained in the current study is supportive of the aforementioned findings. Prevailing high cost of the β_2_m test has limited it being used as a biomarker in the diagnosis or monitoring of nephropathies in the local setup.

Food consumption study revealed that the majority of the subjects, among both CKDu patients and non-CKDu subjects consumed rice, for all three meals. The variety of rice, the majority consumed, was mostly white raw rice. Red raw rice and parboiled rice, were also eaten, but to a lesser extent by both groups. Out of the tested meals, approximately 91.0% meals of CKDu patients, and 95.8% meals of the non-CKDu subjects, included rice as the staple food. Further, more than 90% of CKDu patients and non-CKDu subjects indicated that rice consumed by them is grown in their own paddy fields. Their preference for white raw rice was linked to its trouble-free availability. Rice grown in their own paddy fields is harvested and milled in local rice mills. The convenience in processing, avoiding parboiling efforts had made them consume, white raw rice over many years, perhaps throughout their lifetime. When the percentages of main meals, including different rice varieties were compared, between the two groups, the pattern of variation was almost the same. There were slight differences between the percentages of other food categories eaten as staple foods, including bread, wheat and rice flour products. Consumption of rice was significantly high (p < 0.05) when compared with the reference food bread and wheat flour products, but the percentage of meals of non-CKDu subjects (96.3%) recorded with rice and rice flour products were higher than that of CKDu patients (91.6%) (Table 4). Therefore, it could be interpreted that rice is less likely to contribute towards the development of CKDu. The low consumption of rice and rice flour products by the CKDu patients compared to non-CKDu subjects may be attributable to loss of appetite in these patients as a result of accumulating uremic substances in blood in the latter stages of CKDu. The percentages of reported main meals with millet and pulses and seeds with CKDu patients and normal subjects were significantly less than that of the reference food. Therefore, their consumption could not be tied up with the development of CKDu.

There’s a hypothesis that heavy metals in rice, although not present at toxic levels, may also contribute towards the etiology of CKDu, when consumed over a long period of time, due to its accumulation in the body [5,7]. However, the frequency of rice consumption between CKDu patients and normal subjects in this study were approximately equal. Hence, evidence to support the above hypothesis is limited from the current study. However, a detailed quantitative analysis of food intake may be necessary, before coming to a definite conclusion on this matter. There might be individual variations in the amount of rice consumed. Generally males consume more food than females, especially those males engaged in manual labour, like farming. High incidence of severe stages of CKDu reported among males [5] could be assumed to be due to their higher consumption. But the unaffected normal subjects consuming rice grown in the same area remains questionable. Though rice alone might not be a contributory factor towards the development of CKDu, long term consumption of contaminated rice with other risk factors including life style, exposure to environmental toxins, genetic susceptibility and selenium deficiency could be augmenting the incidence in certain individuals while sparing others. Analysis of rice consumed by CKDu patients and non-CKDu subjects, for heavy metals, combined with the quantitative analysis of rice intake, would provide a solid background for assessing the effect of heavy metals ingested through rice, on CKDu. The newly introduced hybrid varieties of rice: like the common white raw rice eaten is the 3.5 months variety and red rice is the 3.0 months variety; are harvested within a short period of time. If rice is a contributor towards the etiology of CKDu, then a longer time taken for harvesting may increase the risk of promoting such a condition.

With the objective of finding any significant variation among the consumption of locally grown food items, by CKDu patients and normal subjects, they were questioned about consumption of these items. Green gram, cowpea like pulses and a wide variety of other vegetables were found to be grown by some on a small scale and by a few on a larger scale. Certain food accompaniments like jack fruit, siyambala (*Tamarindus indica*) and elabatu (*Solanum melongena*) were grown in and around their compounds.

In assessing the effect of accompaniments of the main meals on CKDu, other vegetable accompaniments which consisted of roots and tubers which were not grown locally were considered as the reference accompaniment. The most commonly used main meal accompaniment (Table 5), was animal sources in both groups. It included varieties of fish, dry fish, meat and eggs and the recorded number of meals with animal sources was higher with non-CKDu subjects than CKDu patients. This indicates that the effects of accompaniments are more favorable to healthy living than being a risk factor. Though the CKDu patients have been advised to include lesser amount of protein via animal sources in their meals the highest recorded accompaniment in the tested meals were animal sources. However, the consumption was significantly lower than that of the normal subjects and this could be due to facts including CKDu patients following the medical advice and lowering their consumption of animal sources and their appetite being affected by the disease.

When consumption of fruit vegetables was considered, the number of meals reported with CKDu patients was higher than that of non-CKDu subjects and the difference was statistically significant (p < 0.05). Most of the fruit vegetables were grown locally and therefore there is a possibility of them acting as sources containing nephrotoxic agent/s. Analysis of these food items consumed by CKDu patients for possible nephrotoxic agents would enlighten this qualm.

The percentages of meals reported with pulses and products as an accompaniment were almost equal in the two groups. Therefore, though a statistical significance is shown when compared with the reference, a possible risk association could not be justified. Further, out of the pulses and products, lentil was the major one consumed and this was not grown locally.

The meals reported with green leaves were significantly low (p < 0.05) when compared with the reference food, which included other vegetable accompaniments. Therefore, though almost all green leaves were locally grown, a definite relationship with CKD cannot be established. The food items like storage rhizomes of of *N. nucifera* (Nelumbonaceae), is highly available in this region and reported to contain high concentrations of Cd [14]. This study hypothesized that including these rhizomes regularly in the diet, may be a predisposing factor for CKDu. However, none of the tested meals, of both CKDu patients and non-CKDu subjects, included rhizomes of *N. nucifera* as an accompaniment. Though food items like these are much available in the region inhabitants may not be consuming them regularly but exporting to the other parts of the country.

There were limitations encountered in the dietary study. Generally almost all of the subjects consumed 3 main meals per day. However, some were found to have skipped a meal, or taken an extra meal, on the day the dietary survey was conducted. Further, few of the subjects in the normal group, commented that they could not recall what was included in their meals on the previous day. Sometimes second and third visits had to be made, to collect dietary recall data, as on some visits, the study participants were not available. Sri Lankan staple diet is a rice based mixed diet which generally includes rice as the major component and vegetables, green leaves, animal sources etc. in smaller amounts as accompaniments. The choice for accompaniments and the amount of accompaniments consumed by an individual may vary by factors including socioeconomical background and local availability of food. The current study analyzed only the qualitative aspect of the diet of the tested groups, by means of assessing the food types consumed by these populations and number time each food type was reported in the tested meals. A quantitative analysis of the food items consumed by CKDu affected and normal people of the same area would have provided more detail as to quantitative variations among the two groups. The practical hitches as unavailability of food composition tables for cooked local food items, the subjects not been inward patients and their poor compliance in measuring their own food intake at home and training a literate family member for accurate measurement of foods consumed limit the implementing of a quantitative dietary study in the selected populations. The appetite for food could get lessen by the disease among CKDu patients and the current study lacks tracking such defects among the subjects.

There were strengths associated with this type of the dietary study, when repeated 24-hour recalls were made on 3 to 7 occasions [15]. The present dietary study was repeated at 6 month intervals on 3 occasions. The entire dietary study was extended over a period of one and half years, to include all CKDu patients and non-CKDu subjects, and also allowing the capturing of any seasonal or individual variations, that could affect the dietary patterns of the study population.

In conclusion, the types of staple food and their consumption patterns among CKDu patients and non-CKDu subjects were found to be similar. However, dietary studies have not been reported up to date, on CKDu affected populations, in Sri Lanka. Hence, in-depth dietary studies which include the quantitative analysis of dietary intake, total energy and protein intake of the CKDu affected and non-CKDu affected subjects and testing of associated metals in the foods they consume are required. Multifactorial studies assessing all the risk factors associated with CKDu comprehensively and simultaneously would shed more light in revealing in exact mechanism of causation of the disease.

## Conclusions

Urinary β_2_m levels of the spot urine samples of non-CKDu subjects from the same environment as CKDu patients were within the normal limit whilst significant elevation was noted among the CKDu patients. Staple food in diet and the consumption pattern of CKDu patients when compared with non-CKDu subjects did not show any significant variation. Consumption of staple diet alone is less likely to be contributing towards the development of disease, but in conjugation with other risk factors might be making individuals more susceptible to the disease. The difference in level of exposure to plausible factors could have accounted for non-CKDu subjects being unaffected.

## Abbreviations

CKDu: Chronic kidney disease of unknown etiology; β_2_m: β_2_ microglobulin; ELISA: Enzyme linked immunosorbant assay; BMI: Body mass index; Cd: Cadmium.

## Competing interests

The authors declare that they have no competing interests.

## Authors’ contributions

EARIES, PAJP, DBN and RS designed the study, TA performed clinical investigations and selected cases and controls, EARIES performed laboratory investigations, EARIES acquired data, EARIES and KGADW performed the statistical analysis and interpreted the data, EARIES drafted the manuscript, PAJP, DBN, RS revised the manuscript for important intellectual content. PAJP, DBN, RS, TA, EARIES, KGADW read and gave final approval of the version to be published.

## Pre-publication history

The pre-publication history for this paper can be accessed here:

http://www.biomedcentral.com/1471-2369/15/103/prepub
